# Phase II Evaluation of Sensitivity and Specificity of PCR and NASBA Followed by Oligochromatography for Diagnosis of Human African Trypanosomiasis in Clinical Samples from D.R. Congo and Uganda

**DOI:** 10.1371/journal.pntd.0000737

**Published:** 2010-07-06

**Authors:** Enock Matovu, Claire M. Mugasa, Rosine Ali Ekangu, Stijn Deborggraeve, George W. Lubega, Thierry Laurent, Gerard J. Schoone, Henk D. Schallig, Philippe Büscher

**Affiliations:** 1 Department of Veterinary Parasitology and Microbiology, Makerere University, Kampala, Uganda; 2 Department of Biomedical Research, Royal Tropical Institute, Amsterdam, The Netherlands; 3 Department of Parasitology, Institut National de Recherche Biomédicale (INRB), Kinshasa, Democratic Republic of the Congo; 4 Department of Parasitology, Institute of Tropical Medicine, Antwerp, Belgium; 5 Rega Institute, Katholieke Universiteit Leuven, Leuven, Belgium; 6 Coris BioConcept, Gembloux, Belgium; Yale School of Public Health, United States of America

## Abstract

**Background:**

The polymerase chain reaction (PCR) and nucleic acid sequence-based amplification (NASBA) have been recently modified by coupling to oligochromatography (OC) for easy and fast visualisation of products. In this study we evaluate the sensitivity and specificity of the PCR-OC and NASBA-OC for diagnosis of *Trypanosoma brucei gambiense* and *Trypanosoma brucei rhodesiense* human African trypanosomiasis (HAT).

**Methodology and Results:**

Both tests were evaluated in a case-control design on 143 HAT patients and 187 endemic controls from the Democratic Republic of Congo (DRC) and Uganda. The overall sensitivity of PCR-OC was 81.8% and the specificity was 96.8%. The PCR-OC showed a sensitivity and specificity of 82.4% and 99.2% on the specimens from DRC and 81.3% and 92.3% on those from Uganda. NASBA-OC yielded an overall sensitivity of 90.2%, and a specificity of 98.9%. The sensitivity and specificity of NASBA-OC on the specimens from DRC was 97.1% and 99.2%, respectively. On the specimens from Uganda we observed a sensitivity of 84.0% and a specificity of 98.5%.

**Conclusions/Significance:**

The tests showed good sensitivity and specificity for the *T. b. gambiense* HAT in DRC but rather a low sensitivity for *T. b. rhodesiense* HAT in Uganda.

## Author Summary

Diagnosis plays a central role in the control of human African trypanosomiasis (HAT) whose mainstay in disease control is chemotherapy. However, accurate diagnosis is hampered by the absence of sensitive techniques for parasite detection. Without concentrating the blood, detection thresholds can be as high as 10,000 trypanosomes per milliliter of blood. The polymerase chain reaction (PCR) and nucleic acid sequence-based amplification (NASBA) are promising molecular diagnostics that generally yield high sensitivity and could improve case detection. Recently, these two tests were coupled to oligochromatography (OC) for simplified and standardized detection of amplified products, eliminating the need for electrophoresis. In this study, we evaluated the diagnostic accuracy of these two novel tests on blood specimens from HAT patients and healthy endemic controls from D.R. Congo and Uganda. Both tests exhibited good sensitivity and specificity compared to the current diagnostic tests and may be valuable tools for sensitive and specific parasite detection in clinical specimens. These standardized molecular test formats open avenues for improved case detection, particularly in epidemiological studies and in disease diagnosis at reference centres.

## Introduction

Human African trypanosomiasis (HAT) is an important public health problem that affects rural populations of sub-Saharan Africa. Previous estimates indicated an annual incidence of about 70,000 cases [1], [2]. The number of cases in 2006 in the Democratic Republic of the Congo (DRC) and Uganda was recently reported to be 11382 and 486, respectively [3], but given the lack of accurate reporting the actual number of cases is probably higher. The disease in DRC is exclusively linked with *Trypanosoma brucei gambiense* that causes the chronic form of HAT. Uganda represents a unique case as it is the only country reporting both the chronic and the acute form of HAT in hitherto non-overlapping foci. The acute form is caused by *T. b. rhodesiense* and may claim the patients in just a few weeks. Following infection, trypanosomes multiply mainly in the lymph and/or blood (haemolymphatic stage). Over time, the parasites cross the blood-brain barrier and invade the central nervous system (neurological stage). HAT is almost invariably fatal if left untreated and major efforts to control the disease rely on vector and reservoir suppression (both human and animal). For the latter, chemotherapy is the mainstay but unfortunately relies on few drugs with unacceptable toxicity and high relapse rates in some foci [4], [5]. In addition to the search for new trypanocidal compounds, current efforts to overcome the problem of drug resistance involve rational use of existing drugs, such as the recently reported nifurtimox-eflornithine combination therapy (NECT) [6].

Control of HAT is challenged by unsatisfactory diagnostics, although they play a central role in the decision to treat affected individuals and in disease control. The card agglutination test for trypanosomiasis (CATT) [7] is extensively used in screening for *Trypanosoma brucei gambiense* although it may miss cases where the infecting trypanosomes do not express the LiTat 1.3 variable antigen type on which it is based [8], [9]. For a similar reason, most *T. b. rhodesiense* infections can not be detected by the CATT. To date, a field applicable screening test for *T. b. rhodesiense* HAT has remained elusive, despite attempts using procyclic trypanosomes [10], [11]. Hence, definite diagnosis is based on microscopic demonstration of trypanosomes in the blood, lymph or cerebrospinal fluid. However, conventional microscopy exhibits a low sensitivity and is therefore often combined with prior parasite concentration such as the haematocrit centrifugation technique (HCT) [12] and the miniature anion exchange centrifugation technique (mAECT) [13]. Despite these innovations, up to 30% of cases are still missed [14] leaving an undetected human reservoir.

Molecular methods for diagnosis of HAT are increasingly gaining attention as possible ways to overcome the problem of low sensitivity of the current parasite detection methods. Recently, two innovative tests for *T. brucei* detection have been developed, the PCR-Oligochromatography (OC) [15] and the NASBA-OC [16]. Both tests are based on nucleic acid amplification followed by simple and rapid detection of the amplified products by dipstick. While PCR-OC amplifies a short sequence within the 18S ribosomal RNA (rRNA) gene by thermal cycling, NASBA-OC is based on isothermal amplification of the 18S rRNA itself. Both tests showed promising diagnostic accuracy during the phase I evaluation studies [15], [16] as well as satisfactory repeatability and reproducibility in a multi center evaluation study (Mugasa *et al.*, submitted). The aim of the presented study was to evaluate the sensitivity and specificity of the two tests in a case control study in Uganda and DRC.

## Methods

### Ethical considerations

Participant recruitment and specimen collection was coordinated by the Institut National de la Recherche Biomédicale (INRB, Kinshasa) in DRC and by Makerere University (Kampala) in Uganda. Ethical clearance for the study was obtained from the relevant institutional ethical committees in DRC (Ministry of Health), Uganda (Ministry of Health) and Belgium (University of Antwerp). Written consent was obtained from the study participants or their parents/guardians in presence of independent witnesses.

### Study participants

During this prospective study carried out between 2006 and 2008, *T. brucei gambiense* HAT patients were recruited from Dipumba hospital in Mbuji-Mayi (Kasai-Oriental, DRC). Healthy endemic control persons were recruited from the same region and from volunteers at the University of Kinshasa (DRC). In Uganda, *T. b. rhodesiense* HAT patients and healthy endemic control persons were recruited at Namungalwe health centre (Iganga district, Eastern Uganda) and Serere health centre (Soroti district, Northeastern Uganda). Individuals were included in the study if 12 years old or more, not in critical condition and if the informed consent was given. A patient was classified as HAT if parasites were observed in the blood, lymph or cerebrospinal fluid (CSF). Both patients in the early (haemolymphatic) and late (neurological) stage were included. Individuals were classified as healthy endemic controls if they had no history of HAT, no clinical signs suggestive for HAT, were negative by CATT on whole blood and if no trypanosomes were observed in the blood.

### Reference tests

The CATT was executed on all participants in DRC and on the endemic controls in Uganda [7]. When a positive CATT result on whole blood was observed, the CATT was repeated with plasma diluted 1/8 as described by Simarro and co-workers [17]. When positive, the individual was subjected to parasitological detection by direct examination of wet smears from lymph node aspirates, Giemsa stained blood smears (only for *T. b. rhodesiense*), the HCT [12] and/or mAECT [13]. Staging of the disease was done by detecting parasites in the CSF by modified single centrifugation [18] and/or by white blood cell (WBC) counting using the Fuchs-Rosenthal chamber or disposable counting chambers (Uriglass, Menarini). Patients with more than 5 cells per µl CSF and/or with parasites in the CSF were considered late stage HAT.

### Index tests

#### Specimen collection and transportation

Two hundred microlitres of anti-coagulated blood was mixed with 200 µl of Angero NA buffer (Mallinckrodt Baker, USA) which allows storage of the blood specimens without loss of nucleic acid quality. Specimens were then transported in a cool-box containing cooling elements from the collection sites to INRB in DRC or Makerere University in Uganda. Upon arrival the specimens were stored in a refridgerator at 4°C for a maximum of two weeks.

#### Isolation of nucleic acids

Nucleic acids of the blood specimens were extracted at the INRB or Makerere University using the method described by Boom et al. [19]. The nucleic acids were eluted in 50 µ1 of Tris-EDTA (TE) and centrifuged at 8000g for 3 minutes where after 35 µ1 of supernatant was stored at −80°C until further analysis.

#### PCR-OC and NASBA-OC

The nucleic acid extracts were analysed with PCR-OC and NASBA-OC between July and October 2008, as described by Deborggraeve *et al.* [15] and Mugasa *et al.* [16] respectively. In brief, DNA or RNA is amplified by PCR or NASBA where after the amplification products are detected by dipstick. Dipstick test results were read after 10 minutes. Nucleic acids extracted from an *in-vitro* culture of procyclic *T. brucei gambiense* (LiTat 1) were used as a positive control. Ultrapure water and the nucleic acid extract of the blood of a naïve volunteer from the Netherlands were used as negative controls. A test was considered positive if a red line was visible at the test line and if the control lines validated the test result [15], [16]. All test results were read by the same three individuals. In case of discrepancy, the result that was the same by two readers was taken as consensus. The executors of the index tests were not blinded to the participant classification and thus the results of the reference tests.

### Data collection and analysis

Data collection, analysis and reporting were done in consideration of the “Strengthening the Reporting of Observational Studies in Epidemiology” (STROBE) statement [20]. The sensitivity and specificity of PCR- and NASBA-OC were calculated from data entered into contingency tables. Sensitivity was defined as the proportion of HAT cases that are positive by the index test and specificity as the proportion of controls that are negative by the index test. Differences in sensitivity and specificity between the two tests were estimated by the Mc Nemar test and differences among centers were estimated with the Fisher exact test. Agreement between the two tests was determined using the kappa index. A kappa index ranges from 0 to 1 and the higher the value the stronger the agreement. All calculations were estimated at a 95% confidence interval (95% CI).

## Results

### Participants

In the study 68 *T. b. gambiense*, 75 *T. b. rhodesiense* HAT patients and 187 healthy endemic controls were recruited (table 1). Out of the 68 *T. b. gambiense* cases, 17 showed parasites in the blood, 28 in the lymph (of which 13 were blood negative), 60 in the CSF (of which 34 were blood and lymph negative). Out of the 75 *T. b. rhodesiense* cases, 73 showed parasites in the blood and 53 in the CSF (of which 2 were blood negative). No lymph node aspirates were examined in *T. b. rhodesiense* cases.

**Table 1:** Human African Trypanosomiasis (HAT) patients and healthy endemic controls participating in the study.

Collection sites	HAT patients				Endemic controls
	Total	Stage I	Stage II	Parasitology Positive blood	
**D.R.Congo**					
Mbuji-Mayi	68	02	66	17	25
Kinshasa	00	00	00	00	97
**Uganda**					
Namungalwe	25	03	22	25	29
Serere	50	07	43	48	36

### Sensitivity and specificity of PCR-OC and NASBA-OC

An overview of the sensitivity and specificities of both index tests on the blood specimens of the participants recruited in the study is presented in Table 2.

**Table 2:** Sensitivities and specificities of the PCR-OC and NASBA-OC on the blood of HAT patients and healthy endemic controls from D.R. Congo (DRC) and Uganda.

		PCR-OC			NASBA-OC		
Participants	Total N°	N° positive	Sensitivity % (95% CI)	Specificity % (95% CI)	N° positive	Sensitivity % (95% CI)	Specificity % (95% CI)
**HAT patients**							
Overall	143	117	81.8 (74.7–87.3)		129	90.2 (84.2–94.1)	
DRC	68	56	82.4 (71.6–89.6)		66	97.1 (90.0–99.2)	
Uganda	75	61	81.3 (71.1–88.5)		63	84.0 (74.1–90.6)	
**Endemic controls**							
Overall	187	6		96.8 (93.2–98.5)	2		98.9 (96.2–99.0)
DRC	122	1		99.2 (95.5–99.9)	1		99.2 (95.6–99.9)
Uganda	65	5		92.3 (83.2–96.7)	1		98.5 (91.8–99.7)

Note. N° = number; CI = confidence interval.

### HAT patients

Trypanosomes were observed in the blood of 90 out of 143 stage I and II patients. The blood of 117 of the 143 was positive by PCR-OC indicating a sensitivity of 81.8% (95% CI of 74.7–87.3%), while we observed an overall sensitivity of 90.2% (95%CI: 84.2–94.1%) for NASBA-OC. Of the 187 healthy endemic controls, 6 showed a positive PCR-OC result and 2 a positive NASBA-OC result yielding an overall specificity of 96.8% (95% CI: 93.2–98.5%) and 98.9% (95% CI: 96.2–99.7%), respectively. While the difference in specificity of both tests was not significant, the Mc Nemar test indicated a significant difference in sensitivity (*P*<0.05).

### *T. brucei gambiense* HAT

Out of the 68 patients from DRC, 56 were positive by PCR-OC on blood indicating a sensitivity of 82.4% (95% CI: 71.6–89.6%). NASBA-OC was positive on blood from 66 of the 68 patients yielding a sensitivity of 97.1% (90.0–99.2%) which is significantly higher than the sensitivity of PCR-OC (*P*<0.05). In only 17 of the 68 patients had parasites been observed in the blood by the reference standard tests. Sixteen out of these 17 patients were positive by PCR-OC (94%, 95% CI: 73%–99%) while all 17 were positive in NASBA-OC (100%, 95% CI: 82%–100%). Of the 47 patients that were parasitologically negative in blood but positive in lymph or CSF, 36 were positive by PCR-OC and 45 with NASBA-OC. Four patients did not undergo microscopic examination of the blood (trypanosomes observed in other tissues) but were all positive by PCR-OC and NASBA-OC.

### *T. brucei rhodesiense* HAT

Of the 75 patients from Uganda, 73 had parasites in the blood and 2 only in the CSF as determined by the reference tests. Sixty one of the 75 patients were positive by PCR-OC (81.3% 95% CI: 71.1–88.5%) and 63 by NASBA-OC (84%, 95% CI: 74.1–90.6%), which is not significantly higher (*P*>0.05). Of the 2 stage II patients with negative parasite detection results in blood, 1 was positive by PCR-OC and both by NASBA-OC.

### Healthy endemic controls

One of the 122 endemic controls from DRC and 5 of the 65 endemic controls from Uganda were positive by PCR-OC. Hence, the specificity of the test was 99.2% (95% CI: 95.5–99.9%) and 92.3% (95% CI: 83.2–96.7%), on the specimens from DRC and Uganda respectively. The NASBA-OC was positive on 1 endemic control from DRC and 1 from Uganda indicating a specificity of 99.2% (95% CI: 95.5–99.9%) and 98.5% (91.8–99.7%) for DRC and Uganda respectively. These 2 endemic controls were also positive in PCR-OC. There was no significant difference in specificities between PCR-OC and NASBA-OC (*P*>0.05).

### Test agreement and differences among centers

Considering all 330 specimens analysed in this study (143 cases and 187 endemic controls), the two tests exhibited a kappa value of 0.85 (95% CI: 0.74–0.95). In DRC, the two tests showed a kappa value of 0.88 (95% CI: 0.74–1.02) and in Uganda a kappa value of 0.8 (95% CI: 0.63–0.96). The Fisher exact test indicated a significant difference (*P*<0.05) between the sensitivity of NASBA-OC on specimens from DRC and from Uganda, and between the specificities of the PCR-OC for the two countries. When we compared results on the specimens collected in the two health centers in Uganda, we observed no significant difference in the specificity of both tests but a significant difference in sensitivity.

## Discussion

This paper reports on the phase II evaluation of *T. brucei* PCR-OC and NASBA-OC, two innovative molecular tests for the diagnosis of HAT [15], [16]. The PCR-OC showed a sensitivity of 82.4% on blood from 68 *T. brucei gambiense* HAT patients from DRC, while the sensitivity on blood from 75 *T. brucei rhodesiense* HAT patients from Uganda was 81.3%. The sensitivity of the test on blood from the Congolese HAT patients is promising, since most patients were in the neurological stage and parasites were observed in the blood of only 17 patients by the reference tests. One of these 17 patients showed a negative PCR-OC result although parasites were detected in the blood by the HCT [12] on 2 capillaries. The detection threshold of the HCT and PCR-OC were estimated at 500 trypanosomes [21] and 5 trypanosomes per ml of blood [15] respectively. As conventional parasite detection is usually highly specific, the negative PCR-OC test result might be due to loss of DNA quality during specimen transport, storage or nucleic acids extraction. Nevertheless, the observation that 36 out of 47 patients whose blood was parasitologically negative were positive by the PCR-OC indicates higher sensitivity of the test compared to conventional parasite detection on blood. The 81.3% sensitivity of the PCR-OC on the 75 *T. brucei rhodesiense* HAT patients was unexpectedly low, given the fact that parasites were observed in the blood of 73 of the 75 patients. Furthermore, *T. brucei rhodesiense* infections are generally linked with acute HAT and higher parasite load in patient blood. The hypothesis that the PCR-OC is less sensitive on *T. brucei rhodesiense* than on *T. brucei gambiense* is unlikely since we expect the DNA target copy numbers in the two subspecies to be in the same range. The observed low sensitivity on the HAT cases from Uganda could have been loss of DNA or DNA quality due to unsuccessful sample storage or DNA extraction. These samples were collected over a period of two years, nucleic acids being extracted and frozen as they were delivered from the rural treatment centers to the central laboratories. DNA quality could have been checked by amplifying a part of the human ß-globin gene [22] but this could be biased by the much higher number of human cells in the specimen. Batch to batch variation of the PCR-OC is implausible since extensive quality control was performed on the test kits prior to dispatch to trial centers. This highlights a weak point of our study, namely the lack of external quality control on a subset of specimens at a central reference laboratory. In addition, test executors were not blinded to the participant classification and we did not apply subspecies-specific PCRs to confirm the presence of *T. b. gambiense* and *T. b. rhodesiense* in the clinical specimens of the HAT cases. Although the HAT patients in Uganda were recruited in *T. b. rhodesiense* areas, infection with *T. b. gambiense* can not be fully excluded since both subspecies are present in this country. Another limitation of the study was that each specimen was tested only once with each index test. However, both assays have proven to be repeatable and reproducible in a multicentre evaluation study comprising 9 different laboratories (Mugasa et al. submitted).

The PCR-OC was positive for one of the 122 endemic controls from DRC and 5 of the 66 endemic controls from Uganda. These might be true HAT cases since the sensitivity of the CATT is not 100%; confirmed *T. brucei gambiense* HAT patients with negative CATT have been reported [8], [9], [23], while an accurate serological test for *T. b. rhodesiense* HAT remains elusive. However, neither DNA contamination during nucleic acid extraction or PCR, nor cross-reaction of the test with DNA from other organisms can be excluded, although such cross-reactions were not observed during the phase I evaluation [15].

The observed higher sensitivity of the NASBA-OC is not unexpected, given that this assay targets the 18S ribosomal RNA (rRNA) while the PCR-OC targets the 18S rRNA gene. It has been documented that the 18S rRNA is present in approximately 10,000 copies, at least 100 times more that the 18S rRNA gene copy number [24]. Indeed, in a study to compare quantitative assays, van der Meide *et al.* [25] observed that the RNA amplifying assays such as NASBA and real time reverse transcriptase PCR detect lower parasite loads compared to real-time PCR. However, in line with the PCR-OC results, the sensitivity of the NASBA-OC on the *T. b. rhodesiense* HAT specimens is surprisingly low and significantly lower than the sensitivity on the *T. b. gambiense* HAT specimens. Given the general high parasite load in the blood of *T. b. rhodesiense* HAT patients, defects in specimen processing, storage and/or transportation are more likely to have contributed to the observed sensitivity than a lower diagnostic performance of the assay for *T. b. rhodesiense*. Although comparisons between foci should be critically made since specimen collection and DNA extraction was done by different persons and in different laboratories, further evaluation studies may clarify the observed discrepancy in sensitivity on both subspecies. The NASBA-OC showed a higher specificity on the endemic controls compared to PCR-OC. This might indicate that DNA contamination during specimen processing is more likely the cause of the low specificity of PCR-OC than the presence of true HAT cases among the endemic controls.

Recently, Lutumba and colleagues estimated the effectiveness of the best current diagnostic algorithm for *T .b. gambiense* HAT at 80% (quantified in terms of the number of lives saved) [26]. Hence, the observed sensitivities of PCR-OC and NASBA-OC are probably higher than each of the current parasite detection tests used alone and could improve this effectiveness. However, one should bear in mind that the PCR-OC and NASBA-OC are not yet an option for routine diagnosis at the primary care level as they require basic molecular biology laboratory facilities [27]. HAT typically affects rural populations in sub-Saharan Africa where health centers most often suffer from infrastructural limitations and thus only apply less sophisticated diagnostic methods. Yet, these standardized molecular test formats can be valuable tools in disease surveillance and epidemiological studies in which specimens are analysed at central reference laboratories. In recent years, the loop-mediated isothermal amplification (LAMP) has emerged as another isothermal amplification technique for the detection of *T. brucei* nucleic acids [28], [29]. The LAMP, being isothermal, is similar to NASBA but amplifies DNA instead of RNA and might thus be less prone to effects of specimen degradation during transport and storage. Although the diagnostic accuracy of LAMP on clinical specimens still has to be proven, the technique is promising and comparative evaluation of NASBA and LAMP on the same patients and controls would be useful. In this study, we could not evaluate the PCR-OC and NASBA-OC for disease staging as CSF specimens were not included in the analysis with the index tests. Hence, further evaluations of the tests for disease diagnosis and staging are required.

In conclusion, the sensitivity and specificity of the PCR-OC and NASBA-OC were successfully evaluated in a case-control study in DRC and Uganda. The tests showed good sensitivity and specificity for *T. b. gambiense* HAT but a rather low sensitivity for *T. b. rhodesiense* HAT in Uganda.

## Supporting Information

Checklist S1 STROBE checklist
(0.10 MB DOC)
