# Supplementary material for: Phase II Evaluation of Sensitivity and Specificity of PCR and NASBA Followed by Oligochromatography for Diagnosis of Human African Trypanosomiasis in Clinical Samples from D.R. Congo and Uganda
Source: PLoS Negl Trop Dis. 2010 Jul 6;4(7):e737. doi: 10.1371/journal.pntd.0000737 (PMC2897845; doi:10.1371/journal.pntd.0000737)
Supplement: Checklist S1 — STROBE checklist (0.10 MB DOC) [file pntd.0000737.s001.doc]

STROBE Statement—Checklist of items that should be included in reports of ***case-control studies***

**Citation**: Matovu E, Mugasa CM, Ekangu RA, Deborggraeve S, Lubega GW, et al. (2010) Phase II evaluation of sensitivity and specificity of PCR and NASBA followed by oligochromatography for diagnosis of human African trypanosomiasis in clinical samples from D.R. Congo and Uganda. PLoS Negl Trop Dis

|  | Item No | Recommendation | | |
| --- | --- | --- | --- | --- |
| **Title and abstract** | 1 | (*a*) Indicate the study’s design with a commonly used term in the title or the abstract  **Page 1, lines 1-2** | | |
|  |  | (*b*) Provide in the abstract an informative and balanced summary of what was done and what was found  **Page 2, lines 30-46** | | |
| Introduction | | | | |
| Background/rationale | 2 | Explain the scientific background and rationale for the investigation being reported  **Pages 4-5, lines 68-112** | | |
| Objectives | 3 | State specific objectives, including any prespecified hypotheses  **Page 5, lines 112-114** | | |
| Methods | | | |  |
| Study design | 4 | Present key elements of study design early in the paper  **Page 6, line 126** | | |
| Setting | 5 | Describe the setting, locations, and relevant dates, including periods of recruitment, exposure, follow-up, and data collection  **Page 6, lines 126-138** | | |
| Participants | 6 | (*a*) Give the eligibility criteria, and the sources and methods of case ascertainment and control selection. Give the rationale for the choice of cases and controls  **Page 6, lines 126-138** | | |
|  |  | (*b*) For matched studies, give matching criteria and the number of controls per case  **Not applicable** | | |
| Variables | 7 | Clearly define all outcomes, exposures, predictors, potential confounders, and effect modifiers. Give diagnostic criteria, if applicable  **Not applicable** | | |
| Data sources/ measurement | 8* | For each variable of interest, give sources of data and details of methods of assessment (measurement). Describe comparability of assessment methods if there is more than one group  **Not applicable** | | |
| Bias | 9 | Describe any efforts to address potential sources of bias  **Not applicable** | | |
| Study size | 10 | Explain how the study size was arrived at  **Pages 6-7, lines 141-150** | | |
| Quantitative variables | 11 | Explain how quantitative variables were handled in the analyses. If applicable, describe which groupings were chosen and why  **Not applicable** | | |
| Statistical methods | 12 | (*a*) Describe all statistical methods, including those used to control for confounding  **Page 8, lines 181-189** | | |
|  |  | (*b*) Describe any methods used to examine subgroups and interactions  **Not applicable** | | |
|  |  | (*c*) Explain how missing data were addressed  **Not applicable** | | |
|  |  | (*d*) If applicable, explain how matching of cases and controls was addressed  **Not applicable** | | |
|  |  | (*e*) Describe any sensitivity analyses  **Page 8, lines 182-185** | | |
| Results | | |  | |
| Participants | 13* | (a) Report numbers of individuals at each stage of study—eg numbers potentially eligible, examined for eligibility, confirmed eligible, included in the study, completing follow-up, and analysed  **Page 8, lines 193-199** | |  |
|  |  | (b) Give reasons for non-participation at each stage  **Not applicable** | |  |
|  |  | (c) Consider use of a flow diagram  **Process of participant selection and testing was limited thus did not require the inclusion of a flow diagram** | |  |
| Descriptive data | 14* | (a) Give characteristics of study participants (eg demographic, clinical, social) and information on exposures and potential confounders  **Not applicable** | |  |
|  |  | (b) Indicate number of participants with missing data for each variable of interest  **Not applicable** | |  |
| Outcome data | 15* | Report numbers in each exposure category, or summary measures of exposure  **Page 8, lines 193-199** | |  |
| Main results | 16 | (*a*) Give unadjusted estimates and, if applicable, confounder-adjusted estimates and their precision (eg, 95% confidence interval). Make clear which confounders were adjusted for and why they were included  **Page 8-10, lines 205-243** | |  |
|  |  | (*b*) Report category boundaries when continuous variables were categorized  **Not applicable** | |  |
|  |  | (*c*) If relevant, consider translating estimates of relative risk into absolute risk for a meaningful time period  **Not applicable** | |  |
| Other analyses | 17 | Report other analyses done—eg analyses of subgroups and interactions, and sensitivity analyses  **Page 10, lines 245-254** | |  |
| Discussion | | |  | |
| Key results | 18 | Summarise key results with reference to study objectives  **Page 10, lines 258-262 and page 11, lines 297-298** | |  |
| Limitations | 19 | Discuss limitations of the study, taking into account sources of potential bias or imprecision. Discuss both direction and magnitude of any potential bias  **Page 11, lines 286-290** | |  |
| Interpretation | 20 | Give a cautious overall interpretation of results considering objectives, limitations, multiplicity of analyses, results from similar studies, and other relevant evidence  **Page 13, lines 347-350** | |  |
| Generalisability | 21 | Discuss the generalisability (external validity) of the study results  **Pages 12-13, lines 326-345** | |  |
| Other information | | | |  |
| Funding | 22 | Give the source of funding and the role of the funders for the present study and, if applicable, for the original study on which the present article is based  **Page 13, lines 354-359** | |  |

*Give information separately for cases and controls.

**Note:** An Explanation and Elaboration article discusses each checklist item and gives methodological background and published examples of transparent reporting. The STROBE checklist is best used in conjunction with this article (freely available on the Web sites of PLoS Medicine at http://www.plosmedicine.org/, Annals of Internal Medicine at http://www.annals.org/, and Epidemiology at http://www.epidem.com/). Information on the STROBE Initiative is available at http://www.strobe-statement.org.
